# New insights on the expression patterns of specific Arabinogalactan proteins in reproductive tissues of *Arabidopsis thaliana*

**DOI:** 10.3389/fpls.2022.1083098

**Published:** 2022-12-02

**Authors:** Diana Moreira, Ana Lúcia Lopes, Jessy Silva, Maria João Ferreira, Sara Cristina Pinto, Sara Mendes, Luís Gustavo Pereira, Sílvia Coimbra, Ana Marta Pereira

**Affiliations:** ^1^ Department of Biology, Faculty of Sciences, University of Porto, Porto, Portugal; ^2^ Laboratório Associado para a Química Verde (LAQV) Requimte, Sustainable Chemistry, University of Porto, Porto, Portugal; ^3^ Biosystems and Integrative Sciences Institute – BioISI, Porto, Portugal; ^4^ Department of Biology, University of Minho, Campus de Gualtar, Braga, Portugal; ^5^ GreenUPorto - Sustainable Agrifood Production Research Centre, Universidade do Porto, Porto, Portugal

**Keywords:** Arabinogalactan proteins, pollen-pistil interactions, female gametophyte, male gametophyte, plant reproduction

## Abstract

Arabinogalactan proteins (AGPs) are hydroxyproline-rich glycoproteins containing a high proportion of carbohydrates, widely distributed in the plant kingdom and ubiquitously present in land plants. AGPs have long been suggested to play important roles in plant reproduction and there is already evidence that specific glycoproteins are essential for male and female gametophyte development, pollen tube growth and guidance, and successful fertilization. However, the functions of many of these proteins have yet to be uncovered, mainly due to the difficulty to study individual AGPs. In this work, we generated molecular tools to analyze the expression patterns of a subgroup of individual AGPs in different Arabidopsis tissues, focusing on reproductive processes. This study focused on six AGPs: four classical AGPs (AGP7, AGP25, AGP26, AGP27), one AG peptide (AGP24) and one chimeric AGP (AGP31). These AGPs were first selected based on their predicted expression patterns along the reproductive tissues from available RNA-seq data. Promoter analysis using β-glucuronidase fusions and qPCR in different Arabidopsis tissues allowed to confirm these predictions. *AGP7* was mainly expressed in female reproductive tissues, more precisely in the style, funiculus, and integuments near the micropyle region. *AGP25* was found to be expressed in the style, septum and ovules with higher expression in the chalaza and funiculus tissues. *AGP26* was present in the ovules and pistil valves. *AGP27* was expressed in the transmitting tissue, septum and funiculus during seed development. *AGP24* was expressed in pollen grains, in mature embryo sacs, with highest expression at the chalazal pole and in the micropyle. *AGP31* was expressed in the mature embryo sac with highest expression at the chalaza and, occasionally, in the micropyle. For all these AGPs a co-expression analysis was performed providing new hints on its possible functions. This work confirmed the detection in Arabidopsis male and female tissues of six AGPs never studied before regarding the reproductive process. These results provide novel evidence on the possible involvement of specific AGPs in plant reproduction, as strong candidates to participate in pollen-pistil interactions in an active way, which is significant for this field of study.

## Introduction

Arabinogalactan proteins (AGPs) are a multifaceted family of hydroxyproline-rich glycoproteins (HRGPs) highly glycosylated by O-linked arabinogalactan (AG) polysaccharides. The protein backbone of AGPs, which constitutes only 2-10% (w/w) of the molecule, is rich in hydroxyproline residues substituted with arabinose, galactose, and other carbohydrates representing 90-98% (w/w) of the molecule (Showalter, 2001; Ellis et al., 2010; Hijazi et al., 2014; Silva et al., 2020). Some AGPs are predicted to have a C-terminal glycosylphosphatidylinositol (GPI) anchor signal sequence which allows them to be anchored to the plasma membrane (Schultz et al., 2002; Borner et al., 2003; Nguema-Ona et al., 2014), and this GPI anchor can be cleaved for AGP release into the cell wall or extracellular environment (Schultz et al., 2002; Borner et al., 2003; Nguema-Ona et al., 2014; Zhou and Dresselhaus, 2019; Beihammer et al., 2020). These two main characteristics of AGPs makes them perfect candidates to be involved in signaling mechanisms in various plant developmental processes (Pereira et al., 2015).

AGPs are divided into different subfamilies: classical AGPs, AG peptides, Lys-rich AGPs, FLAs (Fasciclin-Like AGPs), early nodulin-like AGPs (ENODLs) with plastocyanin-like domains, Xylogen-like AGPs (XYLPs) with non-specific lipid transfer protein (nsLTP) domains, and other chimeric AGPs (Borner et al., 2002; Schultz et al., 2002; Borner et al., 2003; Johnson et al., 2003; Mashiguchi et al., 2009; Ma and Zhao, 2010; Showalter et al., 2010; Kobayashi et al., 2011; Ma et al., 2017). AGPs are spread throughout the plant kingdom, conserved during evolution, and are complex plant macromolecules (Silva et al., 2020) found in the plasma membrane, cell wall, apoplastic space, and secretions, throughout the plant. They are developmentally regulated and play important roles in vegetative and reproductive growth and development, somatic embryogenesis, cell expansion and plasticity, and programmed cell death (Pereira et al., 2016b). Since about 30 years ago, studies on AGPs suggested that they may play major roles in angiosperm sexual plant reproduction. The first studies of these molecules with monoclonal antibodies specific for AGP-specific sugar chains revealed that AGPs are distributed differently within and between different cell types and reproductive tissues (Coimbra et al., 2007).

Nowadays, evidence from studies with different approaches such as immunolocalization, β-Yariv experiments, bioinformatics, reverse and forward genetics, degradation enzymes targeting specific parts of AGPs sugar chains and chemical synthesis of specific sugar chain structures (Coimbra et al., 2007; Pereira et al., 2014; Pereira et al., 2016a; Su and Higashiyama, 2018; Lopes et al., 2019; Silva et al., 2020) greatly supports the involvement of AGPs in somatic embryogenesis (van Hengel et al., 2002; Ellis et al., 2010; Duchow et al., 2016), in female gametogenesis initiation (Acosta-García and Vielle-Calzada, 2004; Demesa-Arévalo and Vielle-Calzada, 2013), pollen grain (PG) development and pollen tube (PT) germination (Coimbra et al., 2009a; Li et al., 2010; Tan et al., 2012; Costa et al., 2013a; Pereira et al., 2016a; Miao et al., 2021), in PT growth and PT targeting to the ovules (Pereira et al., 2014; Hou et al., 2016; Mizukami et al., 2016; Lopes et al., 2019) and in polytubey block (Pereira et al., 2016a; Pereira et al., 2016b).

However, what lies behind the specific function of each of these glycoproteins remains to be discovered. One of the possibilities is that the linkages between AGPs and the other components of the cell wall, mainly hemicelluloses and pectic polysaccharides are essential for their structural functions (Hijazi et al., 2014; Leszczuk et al., 2019; Sanhueza et al., 2022). On the other hand, the fact that these molecules are 90% composed of sugars indicates that their functions may be governed by highly variable glycosylation patterns that constitute each AGP, as Pennell et al. (1991) suggested. Previous studies have revealed the importance of AGP carbohydrates in reproduction (Nguema-Ona et al., 2007; Cannesan et al., 2012). A specific work in *Torenia* demonstrated that AGP carbohydrates are essential for PT capacitation to respond to female signals in this plant. AMOR was identified as a methyl-glucuronosyl arabinogalactan responsible for this function (Mizukami et al., 2016; Jiao et al., 2017). Mutants of AGP-specific Hyp-O-β-galactosyltransferases (GALTs), *galt2, galt3*, *galt4, galt5 and galt6* (Basu et al., 2013; Basu et al., 2015a; Basu et al., 2015b) and *hptg1, hptg2* and *hptg3* (Ogawa-Ohnishi and Matsubayashi, 2015) also confirmed the significance of AGP carbohydrates in their functions making them compelling candidates for mediating cellular communication processes. The quintuple mutant *galt25789* demonstrated aberrant phenotypes related to reproductive processes, namely, defective seed-set and male gametophytic defects (Kaur et al., 2021; Kaur et al., 2022). Furthermore, the ability of calcium ions to bind to AGP sugar residues has been shown to strongly influence their functions (Leszczuk et al., 2019; Lopez-Hernandez et al., 2020). Mutants of glucuronosyltransferases (GLCATs), responsible for the addition of glucuronic acid (GlcA) to the AGP sugar chains were developmentally defective due to the reduced Ca^2+^ binding to GlcA residues of AGPs (Lopez-Hernandez et al., 2020; Zhang et al., 2020). Recently, a review by Lamport et al. (2021) clearly points AGPs as essential players in controlling cytosolic Ca^2+^ levels in the plasma membrane and regulating plant metabolism.

Considering all these aspects, one of the processes where the study of AGPs is very much rekevant, is the reproductive process. Revealing the specific functions and mode of action of these glycoproteins in plant reproduction is of extreme importance to better understand the process leading to successful seed formation in angiosperms. Reproduction in flowering plants entails several molecular players and begins when a PG reaches a pistil’s stigma, and germinates a PT. The PT carries the two sperm cells travelling along the stigmatic cells and the transmitting tract (TT) – rich in exudates containing glycoproteins – to target the ovule, which protects the embryo sac with its female gametes, the antipodals and the synergids, the latest attracting the PT. Near an ovule, PTs turn their growth direction into the ovule to enter it by one of the two synergids. After synergid’s degeneration, sperm cells are released and fuse the egg cell and central cell, developing into the embryo and endosperm, initiating seed development (Higashiyama and Takeuchi, 2015; Dresselhaus et al., 2016; Zhou and Dresselhaus, 2019). The involvement of some specific AGPs in different steps of the reproductive processes, from male and female gametophyte formation to double fertilization and seed formation has already been described (Pereira et al., 2016b; Lopes et al., 2019; Pereira et al., 2021). AGP18, AGP22 and AGP14 are essential for the development of the female gametophyte (Acosta-García and Vielle-Calzada, 2004; Tucker et al., 2012; Demesa-Arévalo and Vielle-Calzada, 2013). AGP6, AGP11, AGP23 and AGP40 are pollen-specific AGPs involved in PGs germination and developmental processes such as endocytosis-mediated plasma membrane remodeling during pollen development (Costa et al., 2013a; Pereira et al., 2016a; Pereira et al., 2016b; Lopes et al., 2019). FLA3 and FLA14 are involved in microspore development, affecting cellulose deposition, and in pollen development and premature pollen germination prevention inside the anthers under high relative humidity conditions in Arabidopsis, respectively (Li et al., 2010; Miao et al., 2021). In rice, *MICROSPORE AND TAPETUM REGULATOR1* (*MTR1*) encodes a FLA essential for tapetum formation and microspore development (Tan et al., 2012). A group of chimeric AGPs (ENODL11/12/13/14/15) is known control PT reception (Hou et al., 2016); and AGP4/JAGGER is important for persistent synergid cell death and cessation of PT attraction into the ovules, being involved in the block to polyspermy (Pereira et al., 2016b).

A first approach to better understand the functions of these complex glycoproteins is to describe their expression patterns in different flower tissues. The expression patterns in the reproductive tissues of one group of AGPs (*AGP1, AGP4, AGP9, AGP12, AGP15*, and *AGP23*) was previously described (Pereira et al., 2014; Pereira et al., 2016a). Almost a decade later, still, many AGPs have their functions and expression patterns unknown, other than the *in-silico* data which have not yet been confirmed experimentally. In this work, we report the expression patterns of another group of AGPs (*AGP7, AGP24, AGP25, AGP26, AGP27* and *AGP31*) using several constructs, complemented with qPCR data, and bioinformatic analysis. These results provide novel evidence supporting the involvement of more AGPs in sexual plant reproduction.

## Material and methods

### Plant materials and growth conditions

*Arabidopsis thaliana* (Columbia-0 ecotype) was obtained from the Eurasian Arabidopsis Stock Centre (NASC). All plants used in this study were grown in soil under long-day conditions (16 h of light at 22°C and 8 h of darkness at 18°C with 50-60% relative humidity and a light intensity of 180 µmol m^-2^ s^-1^. For phosphinothricin acetyltransferase selection, the seedlings were sprayed with 200 mg L^–1^ of glufosinate ammonium (BASTA^®^; Bayer Crop Science) three or four times every 2 days over a 10-day period.

### Construct generation and plant transformation

Genomic regions corresponding to the promoters of six AGP genes (*AGP7, AGP24, AGP25, AGP26, AGP27* and *AGP31*) were amplified using Phusion DNA polymerase (Thermo Scientific), with the primer pairs described in **Supplemental Table 1**. The promoter regions were always amplified from the end of the untranslated region of the most proximal gene upstream of the respective AGP gene sequence until its own start codon. For genes with promoter regions greater than 3000 bp, genomic fragments of approximately 3000 – 3300 bp positioned upstream of the start codon of the AGP gene of interest were amplified. The PCR products were cloned into pENTR™/D-TOPO (Invitrogen) and subcloned into the binary vector pBGWFS7 containing the β-glucuronidase (GUS) gene (Karimi et al., 2002). All constructs were confirmed by DNA sequencing. Expression vectors were delivered into *Agrobacterium tumefaciens* GV3101 (pMP90RK) and were all used to transform *A. thaliana* (Col-0) by the floral dip method (Clough and Bent, 1998).

### Detection of GUS activity

GUS assays were performed overnight on inflorescences with flowers at different stages [stages 11, 12, 14, and 15/16, according to Smyth et al. (1990)], as described by Liljegren et al. (2000). After chemical GUS detection, the samples were incubated in a clearing solution [160 g of chloral hydrate (Sigma-Aldrich), 100 ml of water, and 50 ml of glycerol] and kept at 4°C overnight. The following day, inflorescences were dissected under a stereomicroscope (model C-DSD230; Nikon) using hypodermic needles (0.4 × 20 mm; Braun). The opened carpels and ovules that remained attached to the septum were maintained in a drop of clearing solution and covered with a cover slip. A Zeiss Axio Imager AZ microscope equipped with differential interference contrast optics was used. Images were captured with a Zeiss Axiocam MRc3 camera and processed using the Zen Imaging Software. Floral buds at stage 12 (Smyth et al., 1990) for *pAGP24:GUS* were emasculated and collected 24 h later for GUS detection as described above.

### Co-expression analysis based on RNA-seq data

The RNA-seq data used in this study were previously published by Klepikova et al. (2016). Raw reads were downloaded from the SRA project PRJNA314076. Reads were trimmed using the CLC Genomics Workbench (CLC bio, Denmark) with standard parameters: ‘quality scores – 0.005; trim ambiguous nucleotides – 2; remove 5′ terminal nucleotides – 1; remove 3′ terminal nucleotides – 1; discard reads below length 25’. Trimmed reads were mapped using the RNA-seq mapping algorithm implemented in the CLC software to the reference *A. thaliana* genome (TAIR10) allowing only unique mapping (length fraction = 1, similarity fraction = 0.95).

The values of expression (TPM) for each gene in the RNA-seq dataset were analyzed to calculate the meanexpression across all tissues examined. Genes displaying a mean expression of zero were eliminated from further analysis, as these genes were not expressed in any tissue. A second data set was calculated for all remaining genes and tissues, the normalized expression values - the expression value in a particular tissue minus the mean of expression divided by the mean of expression – defining the expression profile/pattern of each gene. Here, genes with expression profiles containing large values can be compared to those with small values.

To obtain a co-expression matrix, a Δsum was calculated. The normalized expression values of the gene of interest are subtracted, tissue by tissue, from the normalized expression values of all other genes. The sum of the absolute values of these differences from each tissue were calculated for each gene, this is the Δsum. The genes were ranked by the Δsum, whereby the gene of interest will have a Δsum value of zero and those genes with the most similar expression profiles will have the smallest or zero Δsums.

### Sample collection, RNA isolation and cDNA synthesis

A total of 15 samples for RNA extraction were collected from six-week-old plants, representing five tissues [pistil stage 11, pistil stage 12, anther stage 12, pistil stage 14 and silique stage 17, according to Smyth et al. (1990)] from three biological samples. Fifteen pistils from two plants were used for each stage 11 biological replicate, two siliques from one plant were used for each stage 17 biological replicate, 20 pistils from two plants were used for each stage 12 and stage 14 biological replicate and 120 anthers from two plants were used for each stage 12 anther biological replicate. Samples were immediately frozen in liquid nitrogen and stored at -80°C for follow-up experiments. Total RNA was extracted using the RNeasy Plant Mini Kit (QIAGEN, Germany) according to the manufacturer’s protocol. RNA purity and concentration were measured using a spectrophotometer (DS-11 Series Spectrophotometer/Fluorometer; DeNovix, USA). Only RNA samples with absorption ratios of 1.8–2.1 at 260/280 nm and around 2.0 for 260/230 nm were used for further analysis. RNA integrity was evaluated by checking the presence of 25S and 18S ribosomal RNAs bands in a 1% (w/v) agarose gel electrophoresis. Following the manufacturer’s instructions, 400 ng of total RNA from reproductive tissues was treated with DNase I, RNase-free (Thermo Scientific™, USA). cDNA was synthesized using RevertAid First Strand cDNA Synthesis Kit (Thermo Scientific™, USA) and oligo(dT)_18_ primers to initiate the reactions, according to the manufacturer’s guidelines. The cDNA products of the reproductive tissues were diluted to 2 ng/μL with nuclease-free water prior to qPCR.

### Primer design and qPCR analysis

Primers were designed using Primer3 v.4.1.0 (Koressaar and Remm, 2007; Untergasser et al., 2012; Kõressaar et al., 2018) according to the following parameters: annealing preferably on the 3’ end of the transcript, primer length between 18-25 bp, GC content between 30-70%, melting temperature around 60°C, and amplicon size between 100 and 300 bp. The structural aspects of the primers were also a criterion for primer design: primers with G or C repeats longer than three bases, with more than two G or C repeats in the last five bases at the 3’ end, and with long (>4) repeats of any nucleotide were avoided; primers with G and C at the ends were chosen when possible (**Supplemental Table 2**). Primer specificity was confirmed by conventional PCR and electrophoresis on 1% (w/v) agarose gel.

qPCR reactions were performed in a 10 µL final volume containing 5 µL of 2x SsoAdvanced™ Universal SYBR^®^ Green Supermix (Bio-Rad, USA), 0.125 µL of each specific primer pair at 250 nM, 0.75 µL of nuclease-free water, and 4 µL of diluted cDNA template. The reactions were performed in 96-well plates and run on a CFX96 Real-Time System (Bio-Rad, USA) under the following cycling conditions: initial denaturation at 95°C for 30 s, followed by 40 cycles at 95°C for 15 s and 60°C for 30 s, and an additional data acquisition step of 15 s at the optimal acquisition temperature. All reactions were run in three technical replicates and all assays included non-template controls (NTCs). Using three points of a tenfold dilution series (1:10, 1:100 and 1:1000) from the cDNA of wild-type inflorescences, a standard curve was generated to estimate the PCR efficiency of each primer pair using CFX Maestro software v. 2.0 (Bio-Rad, USA). The slope and coefficient of determination (R^2^) were obtained from the linear regression line and the amplification efficiency (E) was calculated according to the formula E = (10^–1/slope^-1) x 100%. The R^2^ value should be higher than 0.980 and the E value should be between 90% and 110%. After completion of the amplification reaction, melt curves were generated by increasing the temperature from 65°C to 95°C, with fluorescence readings acquired at 0.5°C increments. From the melt curve, the optimal temperature for data acquisition (3°C below the melting temperature of the specific PCR product) was determined and the specificity of the primers was confirmed (**Supplemental Table 3**). The sample maximisation method was employed as the run layout strategy, in which all samples for each defined set were analysed in the same run, thus, different genes were analysed in distinct runs (Hellemans et al., 2007). The quantitative cycle (Cq), baseline correction and threshold setting were automatically calculated using CFX Maestro software v. 2.0 (Bio-Rad, USA). The qPCR products were verified using 1% (w/v) agarose gels.

The expression levels of the target genes were quantified in all samples using three reference genes (*RCE1*, *TUA2* and *YLS8*). Relative gene expression was calculated using the 2^-ΔΔCt^ method (Livak and Schmittgen, 2001). Data were statistically analysed using the GraphPad Prism 9 software (www.graphpad.com). For each analysis, the relative expression differences were compared using one-way ANOVA followed by Dunnett’s multiple comparison test. Statistical significance was set at α = 0.05.

## Results

### Co-expression analysis based on RNA-seq data

**Figure 1:**
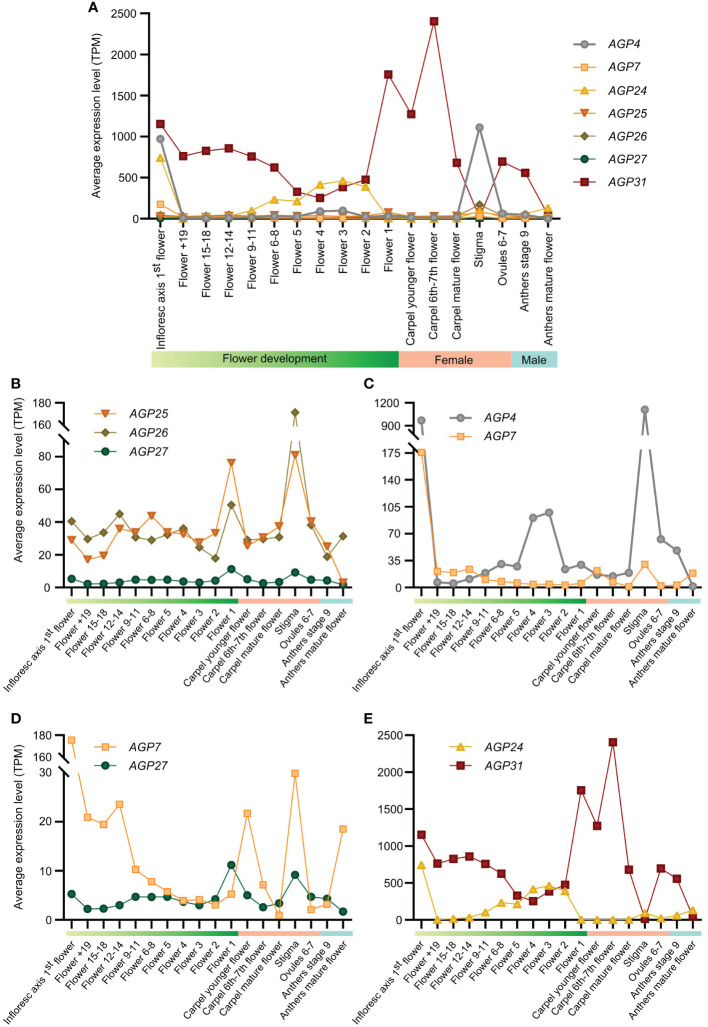
Expression pattern analysis of *AGP4, AGP7, AGP24, AGP25, AGP26, AGP27* and *AGP31* based on RNA-Seq data. Expression levels are presented in transcript per million (TPM) obtained from the RNAseq data published by Keplikova et al. (2016). We show the expression level for the samples most related to the reproductive process [stages according to Klepikova et al. (2016)]. **(A)** Expression pattern analysis based on RNA-seq data for seven AGPs (*AGP4*, *AGP7*, *AGP25*, *AGP26*, *AGP27* and *AGP31*) in samples related to reproductive processes. **(B)** Expression pattern analysis based on RNA-seq data for three phylogenetically close AGPs (AGP25, AGP26 and AGP27) in samples related to reproductive processes. **(C)** Expression pattern analysis based on RNA-seq data for two phylogenetically close AGPs (*AGP4* and *AGP7*) in samples related to reproductive processes. **(D)** Expression pattern analysis based on RNA-seq data for *AGP7* and *AGP27* in samples related to reproductive processes. **(E)** Expression pattern analysis based on RNA-seq data for *AGP24* and *AGP31* in samples related to reproductive processes.

To evaluate whether the expression patterns of different AGPs could be a translation of their phylogenetic relatedness, a co-expression study was performed using a publicly available RNA-seq dataset (Klepikova et al., 2016). We calculated the expression pattern of each AGP regarding all different stages of flower development [from Flower +19 to Flower 1; see **Supplemental Table 4** for correspondence with Smyth et al. (1990) staging], female reproductive structures (carpel at different stages of development, stigma, and mature ovules) and male reproductive structures (anthers at different stages of development). Generally, the AGPs in the study showed a somewhat constant expression level across flower development, with *AGP31* notably having the highest transcript levels (**Figure 1A**). In particular, the expression patterns of the phylogenetically close *AGP25* and *AGP26* (Pereira et al., 2014) were identical showing peaks of transcript abundance in Flower 1 [stage 14 according to Smyth et al. (1990); see **Supplemental Table 4**] and stigma (**Figure 1B**). This was also true for *AGP27*, another AGP closely related to AGP25 and AGP26 (Pereira et al., 2014), however with much lower expression levels. These three AGPs were weakly expressed during the first stages of flower development and in the anthers (**Figure 1B**). Overall, regarding these AGPs, it appears that expression in the female tissues contributes more to the high expression level in the flower, given the higher abundance of transcripts in the female relative to the male tissues (**Figure 1B**).

*AGP7* was previously described as phylogenetically close to *AGP4/JAGGER*, belonging to the same clade (Pereira et al., 2014), which led us to include AGP4 in this bioinformatic analysis, for comparative studies. Surprisingly, these two AGPs showed different expression profiles, with *AGP7* revealing a much lower expression level when compared to *AGP4* (**Figure 1C**). Apart from the inflorescence axis first flower, *AGP4* and *AGP7* transcripts were most abundant in the stigma (**Figure 1C**). In contrast, *AGP7* expression was higher at younger stages of floral development, whereas *AGP4* transcripts were more abundant at later stages (**Figure 1C**). The expression in the male structures was very low for both AGPs. *AGP4* revealed its highest expression at Flowers 3 and 4 (**Figure 1C**), as previously confirmed by promoter analysis with GUS (Pereira et al., 2016b).

Comparing the two AGPs with lowest expression, *AGP7* and *AGP27*, we observed that they had different expression levels, being *AGP7* more abundant at the younger stages of flower development, when compared to *AGP27* (**Figure 1D**). Interestingly, both presented lower expression levels in the mature carpel and a peak of high expression in the stigma (**Figure 1D**). In opposition, while *AGP7* expression in the anthers was reduced, *AGP4* was predicted to be highly expressed in mature anthers (**Figure 1D**). Therefore, despite not being the most phylogenetically related, *AGP7* and *AGP27* expression patterns showed some similarities in stigma, ovules, and Flowers 2-5.

Finally, we compared AGPs with the most distinct expression profiles: *AGP24* and *AGP31* (**Figure 1E**). *AGP24* transcript abundance increased during flower development, whereas *AGP31* expression decreased (**Figure 1E**). *AGP31* was highly expressed in carpels and ovules, while *AGP24* transcripts were abundant in Flowers 2 to 6-8 and were almost absent in the male tissues (**Figure 1E**).

We then decided to take advantage of the RNA-seq data to calculate, relative to the AGPs of interest, which genes shared the most similar expression profiles across the *Arabidopsis* developmental transcriptome (Klepikova et al., 2016). We quantified the difference between the expression profile of the gene of interest and all the genes available in the RNA-seq, which we designated as Δsum (see methods for complete description). Genes with equal expression patterns (the same expression levels across all samples) will show a Δsum of zero. A larger Δsum implies greater differences in the expression pattern (different expression levels in each sample). For these calculations, we considered the flower development samples and female and male structures. This analysis revealed interesting outputs for *AGP24*, *AGP25* and *AGP27*. Several genes putatively co-expressed with *AGP24* were in fact related to reproductive processes. For example, genes highly expressed in pollen, functioning in PT guidance towards the female tissues were found (AT3G13400, AT3G51300, AT2G47040, AT5G14380, and AT2G18470) and genes that might be involved in pollen-pistil interactions, related to calcium homeostasis and receptor-like kinases (AT1G19090 and AT4G38230) (**Supplemental Table 5**). In the case of *AGP25*, we noticed few co-expressed genes that might have important functions in female tissues (AT4G37450/*AGP18*). However, genes expressed in pollen and related to PT growth were found to be co-expressed with *AGP25* (AT2G39900, AT4G08685, AT1G10200, AT4G37450, and AT3G58790) (**Supplemental Table 6**). Notably, for *AGP27*, we found genes possibly related to AGPs glycosylation (AT3G21190 and AT2G22900), one AGP closely related to *AGP27*, *AGP25* (AT5G18690), leucine-rich repeat (LRR) family proteins (AT1G49750), and an Auxin Responsive Factor (ARF; AT2G04850) (**Supplemental Table 7**).

### Differential expression patterns of AGPs in Arabidopsis reproductive tissues

**Figure 2:**
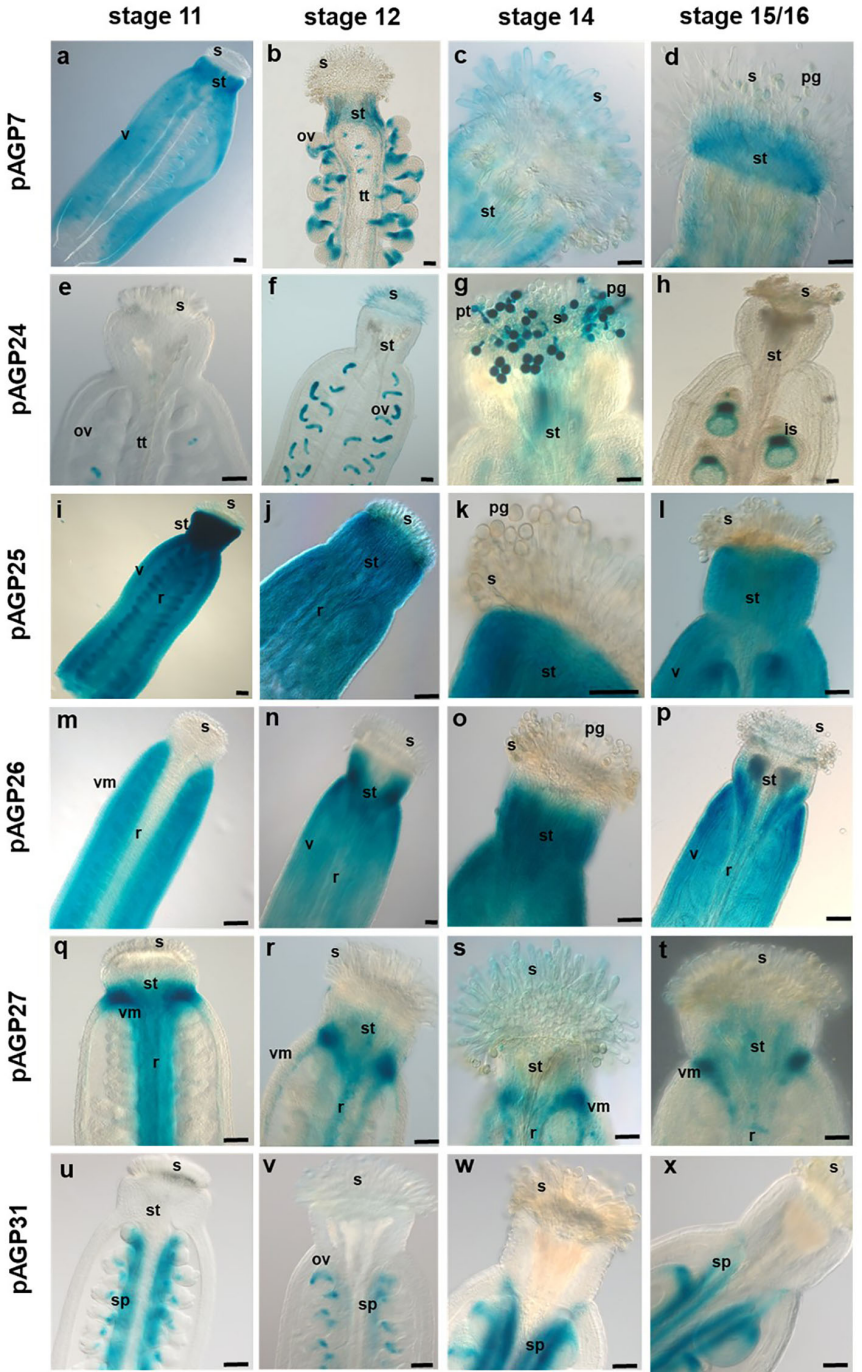
Histochemical localization of GUS activity in transgenic Arabidopsis pistils expressing different *pAGP : GUS* fusion constructs. **(A–D)** GUS activity driven by the *AGP7* promoter at different stages detected in the style (a-d), valves and stigmatic cells **(C)**. **(E–H)** GUS activity driven by the *AGP24* promoter at different stages observed in the ovules **(E, F, H)** and pollen grains **(G)**. **(I–L)** GUS activity driven by the *AGP25* promoter identified in pistil tissues, including the style, valves and replum. **(M–P)** GUS activity driven by the *AGP26* promoter at distinct stages was observed in valves and style. **(Q–T)** GUS activity driven by the *AGP27* promoter visible in the style, replum, valve margins, transmitting tract, and septum. **(U–X)** GUS activity driven by the *AGP31* promoter at different stages visible in the septum and ovules. Flowers of stages 11, 12, 14 and 15/16 (Smyth et al., 1990) were used in this study. ov, ovule; pg, pollen grain; pt, pollen tube; s, stigma; st, style; sp, septum; tt, transmitting tract; vm, valve margin; v, valves; r, replum. Scale bars, 100 μm **(A, B, F, H, I, N)**; 50 μm **(C–E, G, J, L, M, O–V, W, X)**; 20 μm **(F)**.

**Figure 3:**
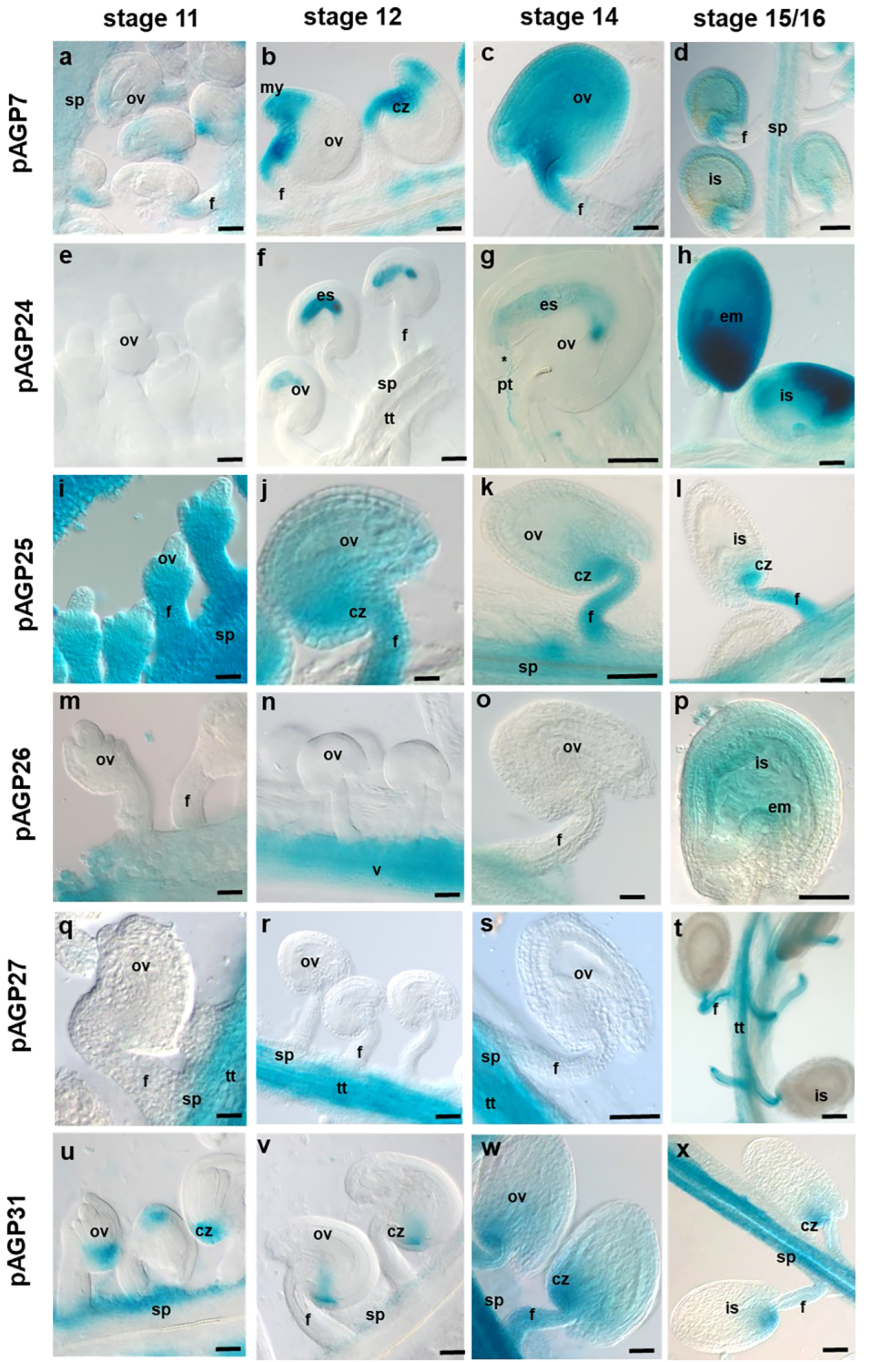
Histochemical localization of GUS activity in transgenic Arabidopsis ovaries expressing different *pAGP : GUS* fusion constructs. **(A–D)** GUS activity driven by the *AGP7* promoter at different stages detected in the chalaza region, micropyle and funiculus **(A, B, D)** and diffuse in all ovules **(C)**. **(E–H)** GUS activity driven by the *AGP24* promoter at different stages, absent in ovules in stage 11 and observed in embryo sac in stages 12 and 14 **(F, G)** and diffused in all immature seeds **(H)**. **(I–L)** GUS activity driven by the *AGP25* promoter was identified in the ovules, including the funiculus, septum and chalaza pole. **(M–P)** GUS activity driven by the *AGP26* promoter at different stages was observed in pistil valves and absent in the ovules; some diffuse expression was detected in stage 16 in immature seeds **(P)**. **(Q–T)** GUS activity driven by the *AGP27* promoter visible in the transmitting tract and septum but absent in the ovule region in stages 11-12 **(Q, R)** and in funiculus in stages14-15/16 **(S, T)**. **(U–X)** GUS activity driven by the *AGP31* promoter in different developmental stages visible in the septum and chalaza pole in stages 11 and 12 **(U, V)** and in funiculus in stages 14 and stage 15/16 **(X, Y)**. Flowers of stages 11, 12, 14 and 15/16 (Smyth et al., 1990) were used in this study. cz, chalaza; f, funiculus; is, immature seed; ov, ovule; pg, pollen grain; pt, pollen tube; s, stigma; st, style; sp, septum; tt, transmitting tract; v, valves. Scale bars, 50 μm **(A–J, L–O, Q, R, T, U, V, W, X)**; 20 μm **(G, K, P, S)**.

The expression patterns of the AGPs selected for this study were analysed at distinct stages (stages 11, 12, 14 and 15/16) of Arabidopsis flower development, according to Smyth et al. (1990), and in different flower tissues (pistil and anthers). Promoter-reporter fusions were constructed for all six AGP genes. The *pAGP7:GUS* fusion-expressing plants revealed an overall weak to moderate GUS activity in pistil tissues at most developmental stages, particularly in the style, valves and stigmatic cells at stage 14 (**Figures 2A–D** and **3A**). However, at stage 12, the *AGP7* promoter led to a very distinct GUS expression pattern in the funiculus, chalazal pole and micropylar region of the ovules (**Figure 3B**). In stage 14, GUS activity spread throughout the entire fertilized ovule (**Figure 3C**). In the later stages of immature seeds (stages 15/16), a clear reduction in the GUS signal was observed (**Figure 3D**).

For *pAGP24:GUS*, a very clear and specific expression signal emerged in the embryo sac and PGs (**Figures 2E–H** and **3E–H**). In stage 14, *pAGP24:GUS* expression in the embryo sac was maintained and was also evident in PTs approaching the micropylar region (**Figure 3G**). To ensure that the GUS activity observed at this stage was not a consequence of PT bursting inside the embryo sac, pistils from *pAGP24:GUS* plants were emasculated at stage 12, and specific GUS activity guided by *AGP24* promoter was confirmed, 2 days after emasculation, in the embryo sac (**Supplemental Figure 1**). In stage 15/16, reporter gene activity spreads throughout the fertilized ovule (**Figure 3H**).

GUS expression driven by *AGP25* promoter was strong in pistil tissues, namely, in the style, valves, replum and ovary, but absent in stigmatic cells at all developmental stages (**Figures 2I–L**). *pAGP25:GUS* plants demonstrated a specific expression in the chalazal pole of the ovule, funiculus and septum at the different developmental stages analyzed (**Figures 3I–L**).

Regarding the plants expressing GUS under the control of *AGP26* promoter, high GUS activity was observed in pistil valves at stage 11 (**Figure 2M**) and from stage 12 onwards, also in the replum and style tissues (**Figures 2N–P**). In ovules, the expression of *AGP26* was absent at stage 11 up to stage 14 (**Figures 3M–O**), but with a diffuse GUS signal present at stage 16, in immature seeds (**Figure 3P**).

For *pAGP27:GUS* plants, GUS activity was found specifically in the style, replum, valve margins, TT and septum in pistil tissues at stage 11 (**Figure 2Q**). From stage 12 onwards, this activity was absent from the replum and detected only at the valve margins, regarding the external pistil tissues (**Figures 2R–T**). From stages 11 to 14, the expression of *AGP27* was specific to the TT and septum, being absent in the ovules (**Figures 3Q–S**). However, at stage 16, GUS activity was detected in the funiculus and TT (**Figure 3T**).

In the case of *pAGP31:GUS* plants, GUS activity was found in the pistil septum tissues at all developmental stages (**Figures 2U–X**). For stages 11 and 12, GUS activity in these plants was found in the chalazal pole of the ovules and septum (**Figures 3U, V**). In stages 14 and 15/16, GUS activity was high at the funiculus, septum and chalazal pole (**Figures 3W, X**).

**Figure 4:**
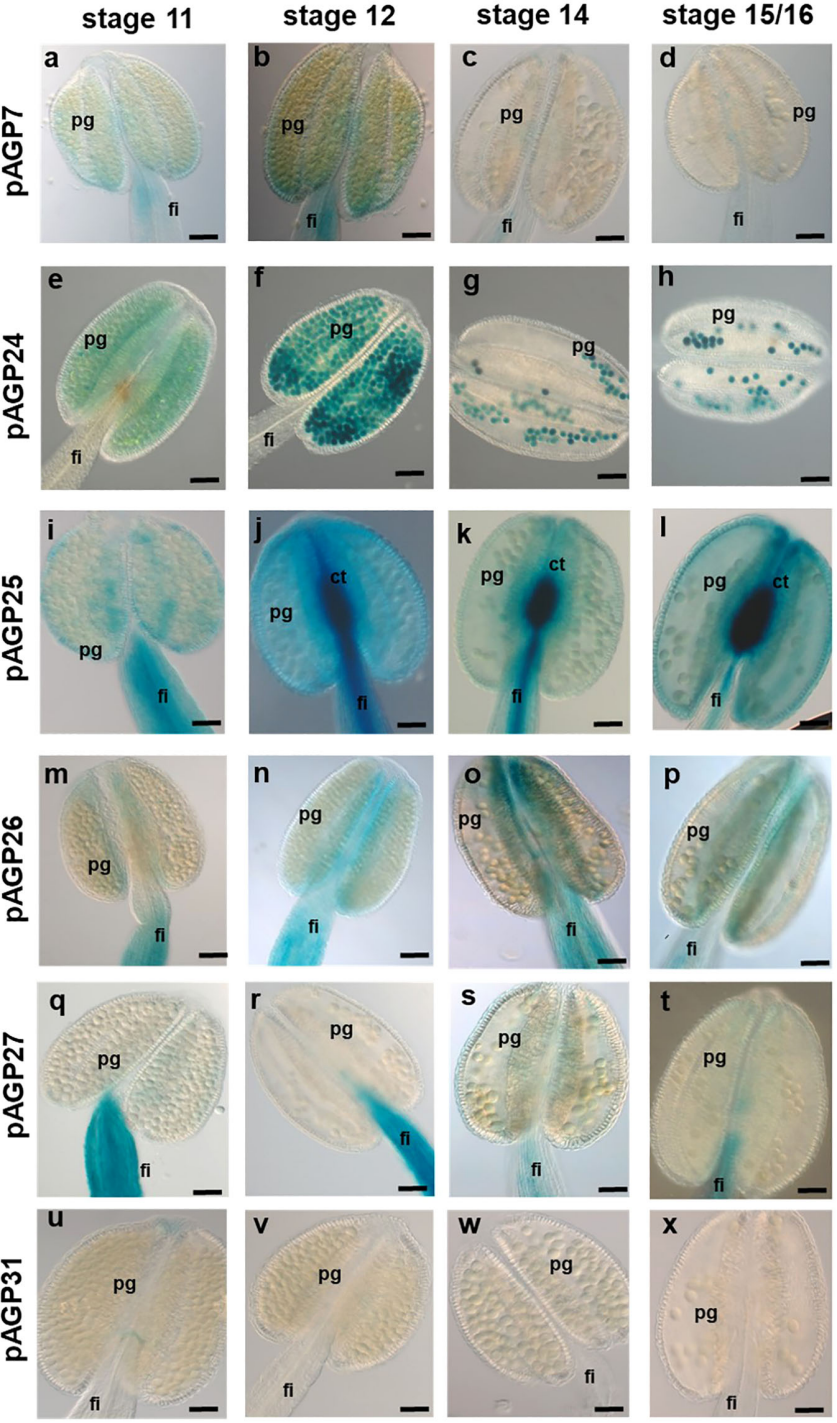
Histochemical localization of GUS activity in transgenic Arabidopsis anthers expressing different *pAGP : GUS* fusion constructs. **(A–D)** GUS activity driven by the *AGP7* promoter at different stages detected only in the anther filament. **(E–H)** Strong GUS activity driven by the *AGP24* promoter is detected in pollen grains at different stages. **(I–L)** GUS activity driven by the *AGP25* promoter was identified in ovules, including the filament and connective tissue. **(M–P)** GUS activity driven by the AGP26 promoter at different stages was observed in anther tissues and filament. **(Q–T)** GUS activity driven by the *AGP27* promoter visible only in the anther filament. **(U–X)** Absent GUS activity driven by the *AGP31* promoter in anther tissues at different stages. Flowers of stages 11, 12, 14 and 15/16 (Smyth et al., 1990) were used in this study. ov, ovule; pg, pollen grain; pt, pollen tube; s, stigma; st, style; sp, septum; tt, transmitting tract; v, valves; ct, connective tissue. Scale bars, 50 μm **(A–V, W, X)**.

In addition to the analysis of female tissues, the expression of these AGPs was also analysed in male tissues (anthers). Regarding *pAGP7:GUS*, GUS activity was present in anther filaments in stages 11 and 12 (**Figures 4A, B**) and completely absent in all other tissues of the anther in the other two developmental stages (**Figures 4C, D**). As for *pAGP24:GUS* plants, GUS activity was very strong in PGs at the different anther developmental stages analyzed (**Figures 4E–H**). In *pAGP25:GUS* plants, GUS signal was detected in the connective tissue of the anther and its filament, but was absent from PGs (**Figures 4I–L**). For *pAGP26:GUS* and *pAGP27:GUS* plants, GUS activity was present only in the anther filaments and totally absent from the remainder of the anther tissues or PGs in all stages of development (**Figures 4M–T**). In *pAGP31:GUS* plants, GUS activity was completely absent from the anther tissues (**Figures 4U–X**).

### AGP gene expression in reproductive tissues

**Figure 5:**
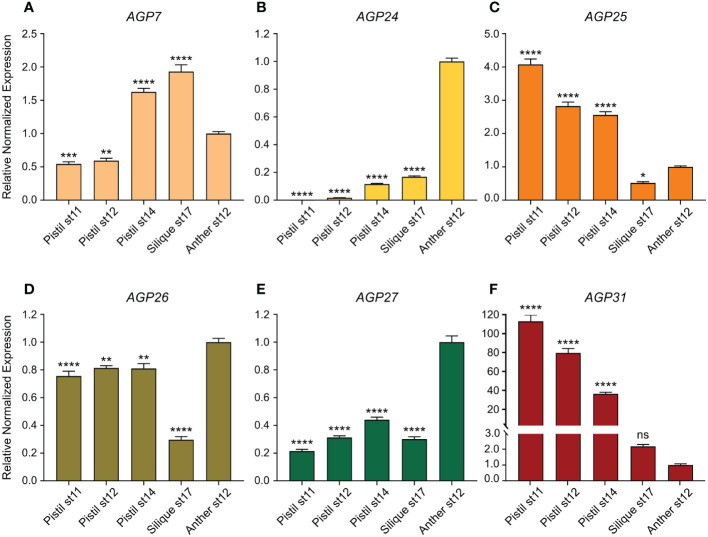
Relative normalized expression levels of *AGP7, AGP24, AGP25, AGP26, AGP27* and *AGP31* in reproductive tissues. **(A)** Relative normalized expression level of *AGP7* in reproductive tissues. **(B)** Relative normalized expression level of *AGP24* in reproductive tissues. **(C)** Relative normalized expression level of *AGP25* in reproductive tissues. **(D)** Relative normalized expression level of *AGP26* in reproductive tissues. **(E)** Relative normalized expression level of *AGP27* in reproductive tissues. **(F)** Relative normalized expression level of *AGP31* in reproductive tissues. The transcript levels were normalized to *RCE1, TUA2* and *YLS8* reference genes. The data correspond to the ratio of the expression compared with anther stage 12 (st12) (Smyth et al., 1990). Error bars represent the standard error of the mean (SEM) of three independent biological replicates, each with three technical replicates. Statistical analyses were performed using a one-way ANOVA followed by Dunnett’s multiple comparisons test. Asterisks indicate value significantly different from anther st12 expression (*, p<0.033; **, p<0.002; ***, p<0.0002; ****, p<0.0001; ns, not significant).

In this study, the relative expression levels of six AGP genes, *AGP7*, *AGP24*, *AGP25*, *AGP26*, *AGP27* and *AGP31*, were quantified by qPCR in distinct reproductive tissues and different developmental stages [pistil stage 11, pistil stage 12, pistil stage 14, silique stage 17 and anther stage 12, according to Smyth et al. (1990)]. The transcript levels were normalized to three reference genes (*RCE1*, *TUA2* and *YLS8*) and are presented relative to anther stage 12 transcript levels, since the main goal was to determine whether the expression of these AGP genes was preferentially expressed in female tissues (**Figure 5A**). In comparison to its expression in anthers stage 12, *AGP7* was downregulated in pistils stages 11 and 12, showing an increase in stages after fertilization, namely pistils stage 14 and siliques stage 17 (**Figure 5B**). The expression levels of *AGP24*, *AGP26* and *AGP27* was lower in female tissues and siliques stage 17 (**Figures 5B, D, E**, respectively), whereas *AGP31* was upregulated in all pistil tissues compared to anthers stage 12 (**Figure 5F**). As for *AGP25*, its expression was upregulated in pistils and downregulated in siliques stage 17 compared to anthers stage 12 (**Figure 5C**).

## Discussion

In this work, we report the expression patterns of six less studied AGPs (AGP7, AGP24, AGP25, AGP26, AGP27 and AGP31), focusing on male and female reproductive tissues. A bioinformatic analysis was also performed to analyse co-expression data for these AGPs. The six AGPs studied revealed specific and differential expression patterns in the three essential reproductive tissues evaluated: whole pistils, ovules, and anthers.

AGP7 is a classical AGP that has never been studied in detail regarding the reproductive process. In this study, *AGP7* expression was shown to be specific of the ovules and funiculus. It was present in the chalaza and micropyle of the ovule before fertilization, and all over the ovule after fertilization. In qPCR analysis, *AGP7* was downregulated in pistils stages 11 and 12 but upregulated in stages after fertilization (at stage 14 and siliques at stage 17). Considering the sequence similarity, described previously in Pereira et al. (2014), and the co-expression study, the specific expression pattern of *AGP7* can be compared to the one of *AGP4/JAGGER*. JAGGER is an important AGP in pollen-pistil interactions being essential for persistent synergid degeneration and polytubey block. *JAGGER* was detected in stigmatic cells, style, TT, funiculus, embryo sac and in the integuments at the micropylar region of the ovule (Pereira et al., 2016b). In some way, both AGP genes revealed a similar expression pattern in the same tissue or adjacent tissues. The sequence similarity between these two AGPs, together with this co-expression data and gene expression pattern results suggest possible redundant roles for JAGGER and AGP7 in pollen-pistil interactions. Interestingly, the polytubey phenotype observed in *jagger* is not fully penetrant, which may be due to the presence of AGP7. Furthermore, these two AGPs are poorly expressed in male structures, indicating that they may be more important for female tissue functions.

*AGP24* was detected by Tucker and Koltunow (2014) in the early stages of ovule development, namely in the functional megaspore, suggesting a possible role in the development of the female gametophyte. In the present study, *AGP24* expression could not be detected in these stages of ovule development, but it was strongly and specifically expressed in ovules before and after fertilization, in the embryo sac and in immature seeds. Surprisingly, *AGP24* was also strongly expressed in PGs and PTs. AGP24 is clearly female and male gametophyte-specific, and its presence in developing seeds is probably a consequence of double fertilization. These results point for possible function of AGP24 during pollen-pistil interactions. *AGP24* revealed lower levels of transcripts in the female tissues compared to the anther tissues by qPCR. This may suggest a more relevant function in the male gametophyte. AGP24 may be acting together with four other AGPs already known to be specifically expressed in male tissues: AGP6, AGP11, AGP23 and AGP40 (Coimbra et al., 2007; Levitin et al., 2008; Coimbra et al., 2009a; Costa et al., 2013a; Pereira et al., 2014). AGP6 and AGP11 are important for PT growth and for preventing early pollen germination (Costa et al., 2013a), and indeed, *AGP6* (AT5G14380) appeared co-expressed with *AGP24* in this analysis. The co-expression data revealed other genes co-expressed with *AGP24*, essential for PT growth such as: *SKU5-SIMILAR 13 (SKS13*; AT3G13400) essential for PT growth across the TT (Zhang et al., 2022); *RHO-RELATED PROTEIN FROM PLANTS 1* (*ROP1*; AT3G51300), that encodes a pollen-specific Rop GTPase, which controls tip growth in PTs (Ménesi et al., 2021; Zhou et al., 2021); *VANGUARD1* (*VGD1*; AT2G47040) encoding a pectin methylesterase that enhances PT growth in female tissues (Jiang et al., 2005); *PROLINE-RICH EXTENSIN-LIKE RECEPTOR KINASE 4* (*PERK4*; AT2G18470), a gene that encodes a member of the Arabidopsis proline-rich extensin-like receptor kinase family that is involved in Ca^2+^ signaling and abscisic acid response in root tip growth (Bai et al., 2009). Other genes from the same family, as *PERK4*, *PERK5* and *PERK12*, were recently shown to be required for proper PT growth, highlighting their role in cell wall assembly and reactive oxygen species homeostasis (Borassi et al., 2021). Nevertheless, *AGP24* expression was also specific of the embryo sac, suggesting an important role in the establishment of the mature female gametophyte and/or interaction between the PT and embryo sac cells. *AGP24* co-expression data showed some interesting genes from other gene families that are important for plant reproduction such as *RECEPTOR-LIKE SERINE/THREONINE KINASE 2* (*RKF2*; AT1G19090) or *CRK1* (*CYSTEINE-RICH RLK*) and *CALCIUM-DEPENDENT PROTEIN KINASE 26* (*ATCPK26*; AT4G38230), belonging to the calcium-dependent protein kinases family (Ca^2+^/CPKs), vital components in the Ca^2+^ signaling pathways. Receptor-like protein kinases (RLKs) are a large family of membrane proteins essential for reproductive development (Cui et al., 2022), and Ca^2+^ is described as an important ion in the entire reproductive process and has also been related to the putative functions and mode of action of AGPs (Cheng et al., 2002; Lamport and Várnai, 2013). A recent study clearly highlighted AGPs as essential players involved in novel molecular pinball machines of the plasma membrane controlling cytosolic Ca^2+^ levels that regulate plant metabolism (Lamport et al., 2021). In addition, recent studies have revealed that the capacity of AGPs to bind Ca^2+^ ions is conferred by GlcA residues in the AGPs sugar chains (Lopez-Hernandez et al., 2020; Pfeifer et al., 2020; Zhang et al., 2020). Therefore, the link between AGPs and Ca^2+^ is very important and probably critical for sexual plant reproduction.

AGP25, AGP26 and AGP27 are a close group of classical AGPs (Pereira et al., 2014) as confirmed by the co-expression studies. However, no specific function for any of these AGPs is known. *AGP25* is strongly expressed in the pistil tissues, septum, ovules and funiculus, and is absent from PGs. qPCR analysis demonstrated that *AGP25* was upregulated in pistils and downregulated in siliques at stage 17, which is consistent with the gradual reduction in GUS activity in ovules from stages 11 to 15/16. *AGP25*, together with *AGP7* and *AGP31* were detected in the chalaza and funiculus tissues. *AGP1*, *AGP12* and *AGP15* are also classical AGPs genes, expressed in the same tissues (Pereira et al., 2014). The chalaza region is essential for nutrient transfer between maternal tissues and the developing embryo (Debeaujon et al., 2000; Ingram, 2010), which may indicate the possible participation of these glycoproteins in nutrition or signaling between the vasculature and the embryo sac (before fertilization), endosperm and/or developing embryo (Pereira et al., 2014). Furthermore, the presence of AGPs along the funiculus could also indicate a possible role of these proteins during the funicular orientation process of the PT until it reaches the embryo sac.

*AGP26* revealed a very diffuse and weak expression pattern in the ovules, showing only a significant expression at the pistil valves and style. *AGP27* showed a very strong expression in the style, TT, septum and in funiculus of developing seeds. However, in the qPCR data*, AGP25* and *AGP27* presented a lower level of transcripts in female tissues and siliques than in the anthers. This may be due to its presence in the filaments of anthers. Given their high amino acid sequence similarity, it was expected that the expression patterns of *AGP25, AGP26* and *AGP27* would be similar, although this was not the case. *AGP25* and *AGP26* were both highly expressed in the pistil tissues and indeed these data are consistent with the co-expression analysis that demonstrated similar expression between *AGP25* and *AGP26*. *In silico* data analysis revealed some genes co-expressed with *AGP25* related to pollen and PT growth such as: *SAH7* (AT4G08685) that encodes a protein similar to pollen allergens; *WLIM2A* (AT2G39900) and *WLIM1* (AT1G10200) encoding members of the Arabidopsis LIM proteins expressed in pollen; and *GALACTURONOSYLTRANSFERASE 15* (*GAUT15*; AT3G58790*)*, belonging to the same family of GAUTs as *GAUT13* and *GAUT14*, which are essential for PT growth (Wang et al., 2013). An interesting AGP was found co-expressed with *AGP25*, *AGP18*, which is essential for female gametogenesis initiation in Arabidopsis (Acosta-García and Vielle-Calzada, 2004; Demesa-Arévalo and Vielle-Calzada, 2013). Regarding *AGP27*, few genes related to the reproductive process were found in the co-expression analysis. In addition to AGP25, one of the AGPs with phylogenetic proximity to AGP27, another two interesting genes were found, one belonging to the LRR family (AT1G49750) and the other to the auxin-responsive gene family (AT2G04850). The LRR family is an important family of proteins in the reproductive process. LRR kinase-like receptors (LRR-RLKs) such as MALE DISCOVERER 1 (MDIS1), MDIS1-INTERACTING RECEPTOR LIKE KINASE (MIK1, MIK2) (Wang et al., 2016) and POLLEN RECEPTOR-LIKE KINASE 6 (PRK6) have been identified as LURE1 receptors on PTs (Takeuchi and Higashiyama, 2016). On the other hand, auxin is a well-known critical plant hormone regulating reproductive development in flowers and participates in the apical–basal patterning of the gynoecium. *AGP27* is expressed along the valve margins and pistil septum, as well as in the upper style, at earlier developmental stages, like *PIN1*, an auxin efflux facilitator involved in auxin transport (Ojangu et al., 2018).

AGP31 is a chimeric AGP whose function has not yet been described in reproduction in Arabidopsis. A previous study showed *AGP31* expression in the vascular bundle throughout the plant and in pistils but not in the stigma (Hijazi et al., 2012). In this study, we confirmed the expression of this AGP in pistil tissues, but it was restricted to the septum and ovules (chalaza and funiculus). *AGP31* transcripts were upregulated in all pistil tissues compared to anthers, which is in accordance with the *pAGP31:GUS* studies.

This work revealed new insights regarding six AGPs (AGP7, AGP24, AGP25, AGP26, AGP27 and AGP31) in the reproductive processes. These AGP genes are expressed in a tissue-specific or developmental stage-specific manner during female reproductive development, and the expression profiles were confirmed by qPCR. *AGP24* was the only gene expressed in the male gametophyte, suggesting its involvement in pollen development and/or pollen-pistil interactions.

This study suggests that some AGPs, such as AGP25/AGP26/AGP27 and AGP7/JAGGER, may have redundant roles. The analysis of multiple order mutants for these AGPs by classical techniques or gene editing techniques, such as CRISPR/Cas9 (Moreira et al., 2020) may help to unravel the function of these glycoproteins abundantly expressed in Arabidopsis reproductive tissues. RNA-seq data analysis allowed us to identify new potential interactors for AGPs as they are co-expressed in the same tissues.

AGPs have been studied for decades; however, most of their functions seem impossible to decipher. Our results clearly showed that specific AGPs are amply present in Arabidopsis reproductive tissues, providing new molecular tools to help dissecting their functions. The “world of AGPs” seems to be an ever-increasing endless one, with many questions arising but few being answered. It is unquestionable the need to study the functions, biosynthesis, and glycosylation of these AGPs.

## Data availability statement

The datasets presented in this study can be found in online repositories. The names of the repository/repositories and accession number(s) can be found in the article/**Supplementary Material**.

## Author contributions

DM and AP designed the research. DM performed the experiments, analyzed the data, and wrote the manuscript. AP designed and obtained the pAGP : GUS constructs. DM, AL, SM conducted the microscopy analyses. SP and SM conducted the bioinformatic analysis. JS, SP and MF conducted the qPCR analyses. LP and SC assisted in interpreting the data and in revising the manuscript. AP conceived the study and helped in reviewing and writing the manuscript. All authors have read and approved the manuscript. All authors contributed to the article and approved the submitted version.

## Funding

This work received finantial support from PT national funds (FCT/MCTES, Fundação para a Ciência e Tecnologia and Ministério da Ciência, Tecnologia e Ensino Superior) through the project UIDB/50006/2020.

## Acknowledgements

We acknowledge Dr Neil Shirley (University of Adelaide, Australia) for valuable assistance with RNA-seq data processing. DM’s research was supported by an FCT PhD grant SFRH/BD/143557/2019. ALL’s research was supported by an FCT PhD grant SFRH/BD/115960/2016. MJF's research was supported by an FCT PhD grant SFRH/BD/143579/2019. JS has received funding from “la Caixa” Foundation (ID 100010434), under the agreement LCF/BQ/DR20/11790010. SCP’s research was supported by an FCT PhD grant SFRH/BD/137304/2018. SC’s research has received funding from an FCT SeedWheels FCT Project - POCI-01-0145-FEDER-027839.

## Conflict of interest

The authors declare that the research was conducted in the absence of any commercial or financial relationships that could be construed as a potential conflict of interest.

## Publisher’s note

All claims expressed in this article are solely those of the authors and do not necessarily represent those of their affiliated organizations, or those of the publisher, the editors and the reviewers. Any product that may be evaluated in this article, or claim that may be made by its manufacturer, is not guaranteed or endorsed by the publisher.
