# Supplementary material for: New insights on the expression patterns of specific Arabinogalactan proteins in reproductive tissues of Arabidopsis thaliana
Source: Front Plant Sci. 2022 Dec 2;13:1083098. doi: 10.3389/fpls.2022.1083098 (PMC9755587; doi:10.3389/fpls.2022.1083098)
Supplement: Supplementary file 1 [file Table_1.docx]

**Supplemental Table 1.** Primer list for AGP promoter amplification and use in GUS fusions.

| **Name** | **Locus** | **Primers (5’ – 3’)** |
| --- | --- | --- |
| ***AGP7*** | AT5G65390 | F - CACCCTGACAAGCTCTAGCTCTCT |
|  |  | R - CCACTCTCTCTAAAGATTTGTT |
| ***AGP24*** | AT5G40730 | F - TGTTTTCTTAATTTCCACGGTG |
|  |  | R - GGTTTTTTCTTTTGATGAATGAG |
| ***AGP25*** | AT5G18690 | F - TGACGCTGAGCTGTAGATTTTG |
|  |  | R - CTCTATCTACTGATCGGAGTGC |
| ***AGP26*** | AT2G47930 | F - ATTCGAAAAGGCGTCCGCAG |
|  |  | R - TTACTGTGAATGTCTCTCCTG |
| ***AGP27*** | AT3G06360 | F - TAGGTGAAGTTTCGATTAGGCG |
|  |  | R - GTTACTGTAATGATGAACAAATC |
| ***AGP31*** | AT1G28290 | F - CAATCAATCACTAATGAACTACCATGT |
|  |  | R - TTTGTTTTGTTTTTGGGTTAGTGA |

| **Supplemental Table 2**. List of target and reference genes and primer sequences used in the qPCR analysis. | | | | |
| --- | --- | --- | --- | --- |
| **Locus** | **Gene Symbol** | **Gene Name** | **Primer Sequences**  **Forward and Reverse (5' - 3')** | **Amplicon length (bp)** |
| *AT5G65390* | *AGP7* | *Arabinogalactan protein 7* | ACTACCTCATGCCTCGCTCA | 158 |
|  |  |  | GCTGAAGACGGAGGAGAAGA |  |
| *AT5G40730* | *AGP24* | *Arabinogalactan protein 24* | TTTGTCTATTGGCGACGATG | 133 |
|  |  |  | ATAGCCAAGACGGTGAAC |  |
| *AT5G18690* | *AGP25* | *Arabinogalactan protein 25* | ACGAACGTTTTGGTTTTCACG | 141 |
|  |  |  | TCAAGAGGGGCAAACACGAC |  |
| *AT2G47930* | *AGP26* | *Arabinogalactan protein 26* | CAAGCGAAATGTCTCCTTCC | 257 |
|  |  |  | TGCTGCTTACTCGCTGTTTC |  |
| *AT3G06360* | *AGP27* | *Arabinogalactan protein 27* | TCCGCAGATCCTGATCCCGCC | 147 |
|  |  |  | TGAAACGGCAAGAAGCCGGAGC |  |
| *AT1G28290* | *AGP31* | *Arabinogalactan protein 31* | CCCCTTCTCTAGCTCCTGCT | 150 |
|  |  |  | GTGGAGACACTGGTGCCTTA |  |
| *AT4G36800* | *RCE1* | *RUB1 CONJUGATING ENZYME 1* | CGGTGGATATGTCGGTCAG | 135 |
|  |  |  | AACGAGGGTCCTTGAGAAAGAG |  |
| *AT1G50010* | *TUA2* | *TUBULIN ALPHA-2 CHAIN* | CATTGAGAGACCCACCTACACC | 78 |
|  |  |  | AACCTCAGAGAAGCAGTCAAGG |  |
| *AT5G08290* | *YLS8* | *YELLOW-LEAF-SPECIFIC GENE 8* | AAGATCAACTGGGCTCTCAAGG | 141 |
|  |  |  | TGGGAAGCTCGATTAGTAACGG |  |

| Supplemental Table 3. Amplification efficiencies, correlation coefficients (R^2^), slope and melting temperatures of qPCR primers of target and reference genes. | | | | |
| --- | --- | --- | --- | --- |
| Gene Symbol | **Efficiency (%)** | **R^2^** | **Slope** | **Melting temperature (°C)** |
| *AGP7* | 107.7 | 0.989 | -3.150 | 88.5 |
| *AGP24* | 100.7 | 0.985 | -3.306 | 88 |
| *AGP25* | 100.2 | 0.998 | -3.316 | 77 |
| *AGP26* | 101 | 0,999 | -3,299 | 84 |
| *AGP27* | 112,1 | 0,989 | -3,063 | 85 |
| *AGP31* | 100,1 | 0,999 | -3,531 | 85 |
| *RCE1* | 96.6 | 0.999 | -3.406 | 81 |
| *TUA2* | 104.4 | 0.999 | -3.221 | 80.5 |
| *YLS8* | 90 | 0.996 | -3.587 | 82.5 |

**Supplemental Table 4.** Description of the samples in Klepikova et al., 2016 compared to stages of flower development in Smyth et al., 1990.

| Klepikova *et al.,* 2016 | Smyth *et al.,* 1990 |
| --- | --- |
| Flower 1 | Stage 14 |
| Flower 2 | Stage 13 |
| Flower3 | Stage 12 |
| Flower 4 | Stage 11 |
| Flower 5 | Stage 10 |
| Flower 6-8 | Stage 8-9 |
| Flower 9-11 | Stage 6-7 |
| Flower 12-14 | Stage 4-5 |
| Flower 15-18 | Stage 3-4 |
| Flower 19^+^ | Stage 3 |
| Inflorescence axis 1^st^ Flower | Stage 3-8 |

**Supplemental Table 5.** The top 24 genes with the most similar expression patterns to *AGP24* based on Keplikova et al. (2016) RNA-seq data. Table is arranged in ascending order according to Δsum value, to indicate genes with most similar pattern. Genes with potentially interesting functions in reproduction are highlighted in bold.

| Δsum | Description | Name | AGI code |
| --- | --- | --- | --- |
| **0.000** | **Arabinogalactan protein 24** | **AGP24** | **AT5G40730** |
| 1.820 | Potassium transporter | **-** | AT5G14890 |
| 2.085 | Encodes PI4Kc3, localizes to the nucleus and has autophosphorylation activity, but no lipid kinase activity. Overexpression mutants display late-flowering phenotype | AtPI4Kgamma3 | AT5G24240 |
| 2.183 | Encodes a pollen/pollen tube-specific gene that is essential for pollen tube growth in the transmitting tract by mediating the biosynthesis of jasmonate that modifies the components of pollen tube cell walls. The SKS13 protein was distributed throughout the cytoplasm and pollen tube walls at the apical region | **SKU5-SIMILAR 13 (SKS13)** | **AT3G13400** |
| 2.415 | Proline-rich extensin-like receptor kinase 4. Functions at an early stage of abscisic acid signalling inhibiting primary root cell elongation by perturbing Ca^2+^ homeostasis | **PROLINE-RICH EXTENSIN-LIKE RECEPTOR KINASE 4 (PERK4)** | **AT2G18470** |
| 2.456 | Pectate lyase family protein | **-** | AT3G01270 |
| 2.525 | Encodes a pollen-specific ROP GTPase, member of the Rho family of small GTP binding proteins that interacts with RIC3 and RIC4 to control tip growth in pollen tubes. These three proteins promote the proper targeting of exocytic vesicles in the pollen tube tip. ROP1 activity is regulated by the REN1 GTPase activator protein | **RHO-RELATED PROTEIN FROM PLANTS 1 (ROP1)** | **AT3G51300** |
| 2.559 | Tightly connected with MAPK signalling to fine-tune stomatal production and patterning | MAPK SUBSTRATES IN THE STOMATAL LINEAGE 3 2 (MASS3) | AT5G20100 |
| 2.572 | Encodes a exopolygalacturonase | POLYGALACTURONASE 4 (PGA4) | AT1G02790 |
| 2.625 | Ribosomal protein l18ae family | **-** | AT3G14595 |
| 2.672 | Share high homologies with a group of pectin methylesterases (PME), pollen specific, and is required for enhancing the growth of pollen tube in style and transmitting tract tissues. | **VANGUARD1 (VGD1)** | **AT2G47040** |
| 2.727 | Hypothetical protein | **-** | AT3G19274 |
| 2.753 | Hydroxyproline-rich glycoprotein family protein | **-** | AT2G22180 |
| 2.793 | HCO3- transporter family | **-** | AT4G32510 |
| 2.831 | Receptor-like serine/threonine kinase | **RECEPTOR-LIKE SERINE/THREONINE KINASE 2 (RKF2)** | **AT1G19090** |
| 2.833 | EXS (ERD1/XPR1/SYG1) family protein | **-** | AT3G29060 |
| 2.852 | Encodes an arabinogalactan protein that is expressed in pollen, pollen sac and pollen tube. Loss of AGP6 function results in decreased fertility due to defects in pollen tube growth | **ARABINOGALACTAN PROTEIN 6 (AGP6)** | **AT5G14380** |
| 2.887 | Member of Calcium Dependent Protein Kinase | **CALCIUM-DEPENDENT PROTEIN KINASE 26 (CPK26)** | **AT4G38230** |
| 2.910 | Plant self-incompatibility protein S1 family | **-** | AT5G26060 |
| 2.914 | Alpha/beta-Hydrolases superfamily protein | **-** | AT5G09430 |
| 2.918 | Glycosyl hydrolase family 35 protein | **-** | AT3G44070 |
| 2.938 | 2-oxoglutarate (2OG) and Fe(II)-dependent oxygenase superfamily protein | **-** | AT2G38500 |
| 2.939 | Encodes a 5-inositol-polyphosphate phosphatase, that, *in vitro*, shows some activity against Ins(1,4,5)P3 and PI(3,4,5)P3, but even higher activity against PI(4,5)P2 | INOSITOL-POLYPHOSPHATE 5-PHOSPHATASE 14 (5PTASE14) | AT2G31830 |
| 2.947 | Putative beta-galactosidase (BGAL11 gene) | BETA-GALACTOSIDASE 11 (BGAL11) | AT4G35010 |
| 2.954 | Exostosin family protein | **-** | AT4G16745 |

**Supplemental table 6.** The top 24 genes with the most similar expression patterns to *AGP25* based on Keplikova et al. (2016) RNA-seq data. Table is arranged in ascending order according to Δsum value, to indicate genes with most similar pattern. Genes with potentially interesting functions in reproduction are highlighted in bold.

| Δsum | Description | Name | AGI code |
| --- | --- | --- | --- |
| 0.000 | **Arabinogalactan protein 25** | **AGP25** | **AT5G18690** |
| 2.389 | Encodes a protein, expressed in leaves, with similarity to pollen allergens. The mRNA is cell-to-cell mobile | SAH7 | AT4G08685 |
| 2.519 | Hypothetical protein | - | AT3G55420 |
| 2.858 | Encodes a protein with putative galacturonosyltransferase activity | **GALACTURONOSYL-TRANSFERASE 15 (GAUT15)** | **AT3G58790** |
| 2.882 | Saccharopine dehydrogenase | - | AT5G39410 |
| 3.035 | Bifunctional inhibitor/lipid-transfer protein/seed storage 2S albumin superfamily protein | GLYCOSYLPHOSPHATIDYLINOSITOL-ANCHORED LIPID PROTEIN TRANSFER 14 (LTPG14) | AT2G44300 |
| 3.129 | O-fucosyltransferase family protein | MANNAN SYNTHESIS RELATED 1 (MSR1) | AT3G21190 |
| 3.131 | AGP18 is a lysine-rich arabinogalactan-protein (AGP). It falls into one subclass with AGP17 and AGP19, other lysine-rich AGPs. It is expressed in young leaves, shoots, roots and flowers and is active in the regulation of the selection and survival of megaspores | AGP18 | **AT4G37450** |
| 3.152 | Violaxanthin de-epoxidase located in chloroplast | - | AT2G21860 |
| 3.161 | Encodes an SAM-dependent methyltransferase superfamily protein that has an N-terminal transmembrane domain and a putative methyltransferase domain, DUF248. It is strongly expressed in the vasculature. Overexpression results in increased phloem and xylem in the plant | INCREASED CAMBIAL ACTIVITY (ICA) | AT5G40830 |
| 3.231 | Transmembrane protein | - | AT4G04190 |
| 3.250 | Encodes a member of the Arabidopsis LIM proteins: a family of actin bundlers with distinct expression patterns. WLIM1, WLIM2a, and WLIM2b are widely expressed, whereas PLIM2a, PLIM2b, and PLIM2c are predominantly expressed in pollen. Regulates actin cytoskeleton organization | **WLIM2A (WLIM2a)** | **AT2G39900** |
| 3.287 | Encodes a member of the DREB subfamily A-4 of ERF/AP2 transcription factor family. The protein contains one AP2 domain. There are 17 members in this subfamily including TINY | ETHYLENE-RESPONSIVE TRANSCRIPTION FACTOR 34 (ERF34) | AT2G44940 |
| 3.312 | A component of the chloroplast signal recognition particle pathway that is involved in LHCP targeting. It is downregulated in response to high light. It recognizes the DPLG motif in Lhcb1. The mRNA is cell-to-cell mobile | CHAOS (CAO) | AT2G47450 |
| 3.325 | Encodes MEIOTIC CONTROL OF CROSSOVERS1 (MCC1), a GCN5-related histone N-acetyltransferase. MCC1 appeared to be required in meiosis for normal chiasma number and distribution and for chromosome segregation. Activation tagging line has increased level of histone H3 acetylation | MEIOTIC CONTROL OF CROSSOVERS1 (MCC1) | AT3G02980 |
| 3.342 | Encodes a class I HD-zip (homeodomain-leucine zipper) protein that is a positive regulator of abscisic acid (ABA)-responsiveness, mediating the inhibitory effect of ABA on growth during seedling establishment | HOMEOBOX PROTEIN 5 (HB5) | AT5G65310 |
| 3.372 | Encodes an ionotropic glutamate receptor ortholog, a member of a putative ligand-gated ion channel subunit family | - | AT2G32390 |
| 3.389 | NAD(P)-linked oxidoreductase superfamily protein | - | AT2G21250 |
| 3.432 | Encodes a homeodomain leucine zipper class I (HD-Zip I) protein which is expressed during the seed-to-seedling transition, regulates some of the network nodes, and affects late seedling establishment. Knock-out mutants for *athb13* showed increased primary root length as compared with wild type (Col-0) seedlings, suggesting that this transcription factor is a negative regulator of early root growth, possibly repressing cell division and/or cell elongation or the length of time cells elongate | ATHB13 | AT1G69780 |
| 3.465 | Protein-tyrosine phosphatase-like, PTPLA | - | AT5G59770 |
| 3.523 | Encodes a Ca^(2+)^-dependent calmodulin-binding protein. AtGT2l specifically targets the nucleus and possesses both transcriptional activation and DNA-binding abilities, implicating its function as a nuclear transcription factor | GT-2LIKE PROTEIN (GT2L) | AT5G28300 |
| 3.531 | Encodes a member of the Arabidopsis LIM proteins: a family of actin bundlers with distinct expression patterns. WLIM1, WLIM2a, and WLIM2b are widely expressed, whereas PLIM2a, PLIM2b, and PLIM2c are predominantly expressed in pollen. Regulates actin cytoskeleton organization | **WLIM1 (WLIM1)** | **AT1G10200** |
| 3.573 | Present in transcriptionally active plastid chromosomes. Involved in plastid gene expression. Essential subunit of the plastid-encoded RNA polymerase (PEP). Mediates phytochrome signalling | PLASTID TRANSCRIPTIONALLY ACTIVE 6 (PTAC6) | AT1G21600 |
| 3.604 | Binding protein | LITTLE ZIPPER 2 (ZPR2) | AT3G60890 |
| 3.611 | Glutamyl-trna (Gln) amidotransferase subunit C | DROUGHT AND FREEZING RESPONSIVE GENE 1 (DFR1) | AT5G17460 |

**Supplemental Table 7.** The top 24 genes with the most similar expression patterns to *AGP27* based on Keplikova *et al*. (2016) RNA-seq data. Table is arranged in ascending order according to Δsum value, to indicate genes with most similar pattern. Genes with potentially interesting functions in reproduction are highlighted in bold.

| Δsum | Description | Name | AGI code |
| --- | --- | --- | --- |
| 0.000 | **Arabinogalactan protein 27** | **AGP27** | **AT3G06360** |
| 3.581 | Uridine kinase-like 4 | URIDINE KINASE-LIKE 4 (UCK4) | AT4G26510 |
| 3.647 | Eukaryotic aspartyl protease family protein | - | AT5G07030 |
| 3.686 | Violaxanthin de-epoxidase-like protein | - | AT2G21860 |
| 3.731 | Expansin B3 | EXPANSIN B3 (EXPB3) | AT4G28250 |
| 3.792 | GTP-binding protein-like protein | EngD-2 | AT1G56050 |
| 3.802 | S-adenosyl-L-methionine-dependent methyltransferases superfamily protein | - | AT1G26850 |
| 3.818 | Pentatricopeptide repeat (PPR) superfamily protein | - | AT1G77405 |
| 3.830 | O-fucosyltransferase family protein | **MSR1** | **AT3G21190** |
| 3.843 | AtBS1(activation-tagged BRI1 suppressor 1)-interacting factor 1 | AIF1 | AT3G05800 |
| 3.923 | Arabinogalactan protein 25 | **AGP25** | **AT5G18690** |
| 3.923 | Saccharopine dehydrogenase | - | AT5G39410 |
| 3.949 | Galactosyl transferase GMA12/MNN10 family protein | **MUCILAGE-RELATED10 (MUCI1)** | **AT2G22900** |
| 3.988 | DNA gyrase subunit B | DNA GYRASE B1 (GYRB1) | AT3G10270 |
| 4.001 | S-adenosyl-L-methionine-dependent methyltransferases superfamily protein | - | AT5G40830 |
| 4.015 | Lactate/malate dehydrogenase family protein | mMDH1 | AT1G53240 |
| 4.021 | Binding protein | ZPR2 | AT3G60890 |
| 4.048 | Phosphate transporter 2 | PHT2;1 | AT3G26570 |
| 4.136 | Encodes AtMurE, a homolog of the bacterial MurE that catalyze the ATP-dependent formation of UDP-N-acetylmuramic acid-tripeptide in bacterial peptidoglycan biosynthesis. Localized to plastids. AtMurE is involved in chloroplast biogenesis | ALBINO OR PALE-GREEN 13 (APG13) | AT1G63680 |
| 4.137 | Pyridoxal phosphate (PLP)-dependent transferases superfamily protein | PSAT2 | AT2G17630 |
| 4.166 | Putative plant snare 12 | NOVEL PLANT SNARE12  (NPSN12) | AT1G48240 |
| 4.170 | Suppressor | - | AT4G18070 |
| 4.194 | Leucine-rich repeat (LRR) family protein | - | **AT1G49750** |
| 4.213 | Auxin-responsive family protein | - | **AT2G04850** |
| 4.221 | Molecular chaperone Hsp40/dnaj family protein | DNA J PROTEIN A5 (DJA5) | AT4G39960 |
