# Supplementary material for: New insights on the expression patterns of specific Arabinogalactan proteins in reproductive tissues of Arabidopsis thaliana
Source: Front Plant Sci. 2022 Dec 2;13:1083098. doi: 10.3389/fpls.2022.1083098 (PMC9755587; doi:10.3389/fpls.2022.1083098)
Supplement: Supplementary file 2 [file DataSheet_1.docx]

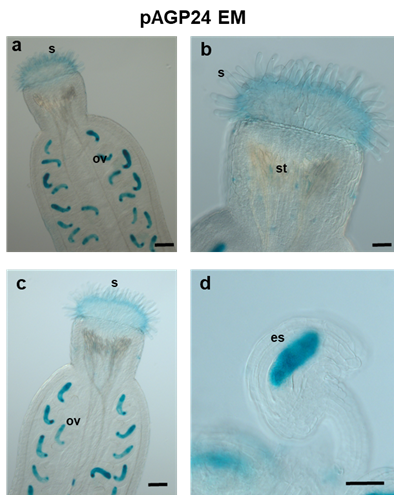


**Supplemental Figure 1. Histochemical localization of GUS activity in transgenic Arabidopsis emasculated pistils expressing the pAGP24:GUS fusion gene.**  **(a-d)** GUS activity driven by the AGP24 promoter in emasculated pistil (without pollen tubes) observed in the ovules. Emasculated pistil stage 12 (Smyth et al., 1990) were used in this study. ov, ovule; s, stigma; st, style; es, embryo sac. Bars, 100 μm (d); 50 μm (a, b, c).
